# Performance Evaluation of State of the Art Systems for Physical Activity Classification of Older Subjects Using Inertial Sensors in a Real Life Scenario: A Benchmark Study

**DOI:** 10.3390/s16122105

**Published:** 2016-12-11

**Authors:** Muhammad Awais, Luca Palmerini, Alan K. Bourke, Espen A. F. Ihlen, Jorunn L. Helbostad, Lorenzo Chiari

**Affiliations:** 1 Department of Electrical, Electronic, and Information Engineering Guglielmo Marconi, University of Bologna, Bologna 40126, Italy; luca.palmerini@unibo.it (L.P.); lorenzo.chiari@unibo.it (L.C.); 2 Department of Neuroscience, Norwegian University of Science and Technology, 7491 Trondheim, Norway; alan.bourke@ntnu.no (A.K.B.); espen.ihlen@ntnu.no (E.A.F.I.); jorunn.helbostad@ntnu.no (J.L.H.); 3 Health Sciences and Technologies Interdepartmental Center for Industrial Research, University of Bologna, Bologna 40126, Italy

**Keywords:** inertial sensors, physical activity classification, overall accuracy, real life conditions, older subjects

## Abstract

The popularity of using wearable inertial sensors for physical activity classification has dramatically increased in the last decade due to their versatility, low form factor, and low power requirements. Consequently, various systems have been developed to automatically classify daily life activities. However, the scope and implementation of such systems is limited to laboratory-based investigations. Furthermore, these systems are not directly comparable, due to the large diversity in their design (e.g., number of sensors, placement of sensors, data collection environments, data processing techniques, features set, classifiers, cross-validation methods). Hence, the aim of this study is to propose a fair and unbiased benchmark for the field-based validation of three existing systems, highlighting the gap between laboratory and real-life conditions. For this purpose, three representative state-of-the-art systems are chosen and implemented to classify the physical activities of twenty older subjects (76.4 ± 5.6 years). The performance in classifying four basic activities of daily life (sitting, standing, walking, and lying) is analyzed in controlled and free living conditions. To observe the performance of laboratory-based systems in field-based conditions, we trained the activity classification systems using data recorded in a laboratory environment and tested them in real-life conditions in the field. The findings show that the performance of all systems trained with data in the laboratory setting highly deteriorates when tested in real-life conditions, thus highlighting the need to train and test the classification systems in the real-life setting. Moreover, we tested the sensitivity of chosen systems to window size (from 1 s to 10 s) suggesting that overall accuracy decreases with increasing window size. Finally, to evaluate the impact of the number of sensors on the performance, chosen systems are modified considering only the sensing unit worn at the lower back. The results, similarly to the multi-sensor setup, indicate substantial degradation of the performance when laboratory-trained systems are tested in the real-life setting. This degradation is higher than in the multi-sensor setup. Still, the performance provided by the single-sensor approach, when trained and tested with real data, can be acceptable (with an accuracy above 80%).

## 1. Introduction

Physical activity (PA) is fundamental for functionality of the human body and it is one of the strong predictors of healthy ageing and wellbeing. Low physical activity in the elderly population is strongly associated with many fall related injuries, age-related loss of muscle, mobility disorders, and loss of independence in daily life. A study conducted by the World Health Organization (WHO) in the 28 member states of European Union (EU), proposed that promotion of physical activity and prevention of falls are among the five priority interventions to promote healthy ageing [1]. The statistics shows that the proportion of falls per year is 30% among the population over 65 which increases to 50% in the population above 80 [1]. Better knowledge about activities of daily life (ADL) is needed in order to design interventions to prevent inactivity and improve health and function during the ageing process.

**Figure 1:**
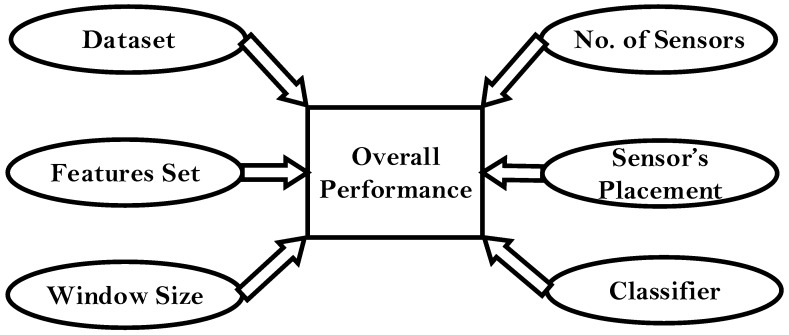
Factors that contribute to the overall performance of the PAC system.

Recent technological advances in the IMU (inertial measurement unit) sensors have encouraged researchers and scientists to incorporate these in personal health systems. This is mainly due to their low cost, low power consumption, small size, wearability, and reliable data transfer capabilities. A typical IMU device is composed of a tri-axial accelerometer and gyroscope capable of measuring linear acceleration and angular velocity. There is an increasing number of physical activity classification (PAC) systems to classify the ADL by utilizing these sensors [2,3,4,5,6,7,8,9,10,11,12,13,14,15,16]. The overall performance of these PAC systems presented in the literature can depend on many factors, illustrated in Figure 1.

(i). **Dataset:** Nature of the datasets differs in terms of the population studied, how and where the ADLs are performed and the type of ADLs included in the dataset. Majority of the existing PAC systems developed in the literature have used datasets collected in a laboratory setting or in a controlled environment with predefined sets of activities [13,14,17,18].
(ii). **Number of sensors:** Varies from a single sensor setup [3] to multiple sensors setup [2,4,5].
(iii). **Placement of sensors:** Varies, covering different body locations in order to record the upper and lower body movements. The common sensor placements are L5, hip, thigh, waist, foot, ankle, chest, and wrist [4,5,14,17,18,19].
(iv). **Features set:** Existing PAC systems are composed of numerous time and frequency domain features, statistical features and bio-mechanical features [8,20].
(v). **Window size:** Window size and overlapping intervals used for the feature computation vary and they may affect the performance of machine learning algorithms and classifiers. The window sizes largely differs across the PAC systems proposed in the literature: 2 s [4], 2.5 s [11], 5 s [5], 5.12 s [3], 6.7 s [2], and 10 s [9]. The overlapping interval used in most of the PAC systems is 50% of the window size [20].
(vi). **Classifier:** In most of the PAC systems, a single classifier is used to differentiate between all the different ADLs in the dataset. A common choice for such classifiers may include a decision tree classifier [2], support vector machine (SVM), artificial neural network (ANN) [13], and K-nearest neighbors (KNN) [4]. However, some systems have attempted to integrate the base level classifiers either by plurality voting [3] or by defining a hierarchical classification process which uses different classifiers for each subset of ADL [6,10,15].

The choice of each single aspect discussed above is crucial in the development of a robust PAC system since all of these factors contribute directly to overall performance. Due to the large diversity in the design process, the existing PAC systems are not directly comparable which hinders the development of new techniques informed by the strengths and the gaps in these systems. Another issue is that most of the existing PAC systems used younger subjects for data collection [3,4,5,6,9,10,13,14,17,21,22] and few systems collected data on older subjects [11,23,24,25,26]. Furthermore, most PAC systems are developed in a controlled environment, which is quite different from real-life conditions [27]. A group of researchers [28] recently proposed a set of recommendations about the standardization of validation procedures for PAC systems in older people, which emphasizes the need to develop and validate the systems using a semi-structured protocol where ADLs are performed in real-life conditions, in addition to the validation performed in the laboratory setting.

In the past, some researchers [10,29,30] have tried to compare the performance of their proposed PAC systems with existing systems. However, in our opinion, they failed to provide a fair comparison, since they did not consider that the factors reported in Figure 1 were just not comparable. Therefore, the present study aims to propose a fair and unbiased benchmark for the field-based validation of existing state of the art (SOA) systems for PAC of older subjects highlighting the gap between the laboratory and real-life conditions. The specific aims of this study are as follows:

(1) To compare the performance of existing PAC systems in a common dataset of activities of older subjects in an unbiased way (i.e., with the same subjects, sensors, sampling frequency, window size and cross-validation procedure), and to investigate the effect of varying window size on system’s performance.
(2) To validate and compare the performance of the PAC systems in real-life scenarios compared to an in-lab setting in order to check if these systems are transferrable to real life settings.
(3) To evaluate the impact of the number of sensors on the performance in the analyses in (1) and (2) using a reductionist approach (i.e., analyzing only the sensing unit worn at the lower back instead of the multi-sensor setup). The lower back location is chosen since it is a very common case that shows no major drawbacks for the monitoring of the activities of older subjects.

For the presented aims, we selected three representative SOA systems for PAC [2,9,10] motivated by the following reasons: (i) diversity in the number of sensors used; ranging from four sensing units by Leutheuser et al. [10] up to six sensing units by Cleland et al. [9]; (ii) use of different time intervals for windowing (ranging from 5 s [10] to 10 s [9]); (iii) different classification techniques i.e., decision tree classifier by Bao et al. [2], SVM by Cleland et al. [9], and hierarchical classification by Leutheuser et al. [10].

Four ADLs (sitting, standing, walking, and lying) are studied in this work in order to provide a fair comparison. These ADLs are chosen as they are the most common in this kind of studies and due to these four activities being present in all of the selected systems.

The rest of the article is structured as follows: Section 2 presents the methodology of the study and the description of the dataset used; in Section 3, results with a comprehensive discussion on the findings are presented; in this section comparative analysis of the three systems is also presented; Section 4, concludes the study.

## 2. Materials and Methods

### 2.1. Data Collection in Real-Life Scenarios

The data collection was performed at the Department of Neuroscience, Faculty of Medicine, at the Norwegian University of Science and Technology (NTNU) Norway, by the research group on Geriatrics, Movement, and Stroke, as part of the ADAPT project (A Personalized Fall Risk Assessment System for promoting independent living). The data collection protocol was composed of two sessions; semi-structured supervised protocol (in-lab) and a free-living unsupervised protocol (out-of-lab). Twenty older subjects (76.4 ± 5.6 years) participated in the study. For both data protocol sessions, video recording was used as a gold standard. Various inertial sensing units were placed on different body locations and a subset of these sensors was used in our analysis: chest, lower back (L5), wrist, waist, thigh, and foot. The details of the sensors used and their respective placements are presented in Table 1. The wrist sensor was down sampled to 100 Hz to keep the same sampling frequency for all sensors. All mentioned sensors were part of in-lab and out-of-lab protocols except the sensor on the feet which was excluded from out-of-lab data recording for usability issues. Each subject performed a variety of ADLs in both sessions with the ADLs analyzed in our study being sitting, standing, walking, and lying. The in-lab session was performed in a smart home environment where subjects were supervised and instructed to perform ADLs. Video recording was performed using the ceiling mounted cameras at 25 fps. The in-lab session was followed by an out-of-lab session on the same day where subjects performed their daily routine activities in an unsupervised way. They were instructed to perform as much ADLs as possible and to incorporate certain tasks into their daily routine. A GoPro camera unit with frame rate of 29 fps (fixed to the chest pointing downward towards the feet) was used to video record the gold-standard information of the ADLs performed in free living protocol. Video annotation of the camera units used in the in-lab and out-of-lab protocol was performed by the recruited raters. Raters were instructed on the marking procedures and activity definitions. For both sessions, video annotation agreement was around 90%. The original sampling frequency (25 Hz) of the annotations was up-sampled to 100 Hz [31]. A detailed description of the ADAPT dataset and the video annotation process is presented in the study protocol by Bourke et al. [31]. Due to technical issues with the wrist sensor, 16 subjects were used for analysis purposes as authenticity of sensed data was compromised in rest of the cases due to missing data at the time of recording. Therefore, four subjects were excluded from the analysis as all selected PAC systems make use of the wrist sensor data.

A summary of the ADLs from 16 subjects analyzed from the in-lab and the out-of-lab protocol is presented in Table 2 and Table 3, respectively. Statistical analysis is performed and various parameters are computed: occurrences (how many times a single ADL occurred in all subjects), mean (average duration of each ADL in seconds), STD (standard deviation of each ADL in seconds), min (minimum duration of each ADL in seconds), max (maximum duration of each ADL in seconds), and range (difference between min and max in seconds).

### 2.2. Implementation of the SOA Systems for PACs Using Their Original Framework

The set of sensors used in our work for the in-lab (S_IN_) and out-of-lab (S_OUT_) analysis performed on the ADAPT dataset is shown in Table 4.

The brief description of the three PAC systems, selected for the comparative analysis is presented in Table 5. It is much evident from Table 5 that all PAC systems possess different solutions for a number of sensors, sensor locations, set of features, classifiers, and time window used for feature computation.

To investigate the sensitivity of the classification accuracy to window size (first specific objective), all systems are trained and tested in the in-lab data with a window size ranging from w = 1 s to w = 10 s in steps of 1 s. The sensor set S_IN_ (Table 4) is used with leave-one-subject-out cross-validation.

Analysis of the out-of-lab data is performed by training and testing all systems with the real-life data. The window size of 5 s is used with the sensor set S_OUT_ (Table 4) and leave-one-subject-out cross-validation is performed. The window size of 5 s is chosen, since it is closer to the window size used by two out of three PAC systems (Table 5).

To address the second specific objective, each PAC system is trained with the in-lab data and tested on the out-of-lab data. To overcome any bias in the training process, the in-lab data of all subjects except one is included in the training stage. The left-out subject is tested in free living conditions (i.e., with the out-of-lab data). In this way, all participants are tested in free living condition using this leave-one-subject-out strategy. The sensor set S_OUT_ is used with the window size of 5 s.

The overlap is set to 50% of the window size for all the analysis. Furthermore, a majority voting scheme is implemented to assign the window labels i.e., if a window of 5 s (500 samples) contains 400 samples of sitting and 100 samples of standing then the assigned label to this window would be sitting.

All of the PAC systems are implemented in MATLAB (Release 2014b, The MathWorks, Inc., Natick, MA, USA) and respective classifiers are implemented using the libraries of Weka data mining software (University of Waikato, Version 3.6.12 [32]). The analysis is performed on a Dell laptop (Model # M3800, Intel^®^ Core™ i7-4712HQ, CPU @2.30Gz, 16GB RAM, 64-bit operating system). For all systems, overall accuracy, accuracy by class and sensitivity by class of all activities is computed in the in-lab training/out-lab testing scenario. The overall accuracy term will be used interchangeably as accuracy or performance in the upcoming sections. The formulas used for the computation of performance metrics are reported in Appendix A and the respective classification methods implemented for each PAC system are described in Appendix B.

### 2.3. Implementation of the SOA Systems for PAC Using a Reductionist Framework

The performance of all systems is also computed in the reductionist framework implemented using only the sensor data collected at waist-level in L5 (third specific objective). The steps in the analysis are the same as described in Section 2.2.

## 3. Results and Discussion

### 3.1. Performance Comparison of the PAC Systems in the In-Lab Setting Using Their Original Framework and Sensitivity Analysis to the Window Size

**Figure 2:**
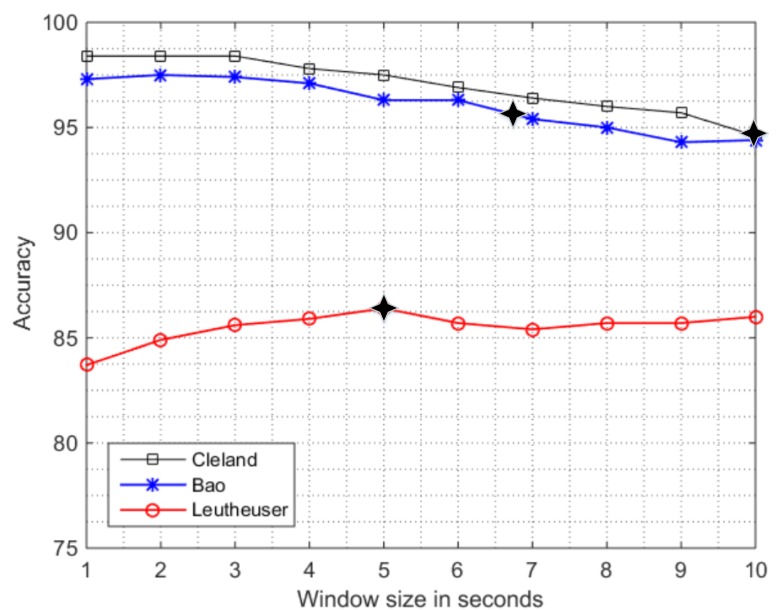
Sensitivity analysis of overall accuracy of in-lab data when window size is increased from w = 1 s to w = 10 s using sensor set S_IN_ (Table 4). The symbol (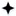) specifies the window size used in the original PAC system by the authors.

Overall accuracy computed for the sensitivity analysis of the in-lab data to different window sizes (w = 1 s to 10 s) is presented in Figure 2. The system by Cleland et al. [9] is the one which performs better in our framework, with an overall accuracy ranging from 98.4% for w = 1 s to 94.6% for w = 10 s. It, hence, shows a degradation by 3.8% when increasing the window size. Our result for in-lab data compares well with the original paper that, for w = 10 s, reported an overall accuracy of 97.3%. The second-best performance we obtained is with the system proposed by Bao et al. [3]. It also shows a decreasing trend in the overall accuracy from 97.3% (for w = 1 s) to 94.4% (for w = 10 s) with a difference of 2.9%. The original system was implemented with w = 6.7 s and had an overall accuracy of 84%; our closest term of comparison is the window with w = 7 s, which produces an accuracy of 95.4%. The accuracy of the system by Leutheuser et al. [10] is fairly below the previous ones. In the system by Leutheuser et al. [10] we obtain an overall accuracy which, unlike previous systems, increases by 2.3%, from 83.7% (w = 1 s) to 86.0% (w = 10 s). Results obtained in our framework (overall accuracy of 86.4%) fits well with the original one at w = 5 s (overall mean classification rate of 89.6%). A possible reason for the increase in the performance (although the performance is the worst of the three) for increasing window sizes of the system by Leutheuser et al. is the difference in the classifier design. Their work is the only one that uses a hierarchical classification approach.

The systems by Bao et al. [2] and Cleland et al. [9] achieved very high accuracies, at the cost of using a large number of sensors, which is a practical issue in real-life conditions. The system developed by Bao et al. uses four sensors and the system proposed by Cleland et al. uses six sensors, which raise feasibility and computational complexity issues for these systems which could make them less practical in real life conditions.

The probable cause in the overall lower performance of the system by Leutheuser et al. could be the fact that in their original implementation six subsets of ADLs were considered (1: HOUSE (vacuuming, sweeping); 2: REST (sitting, standing, and lying); 3: WALK (walking, running, ascending stairs, descending stairs); 4: bicycling; 5: rope jumping; 6: washing dishes). Instead, in our analysis, only two sub-systems are used i.e., REST (sitting, standing, lying) and WALK (walking). The subdivision of ADLs which characterizes this hierarchical classification can be a limitation in implementing the original work when choosing only a subset of activities, as in our case. It could also be an issue if a hierarchical classification approach is implemented on a set of activities which is not the same as the original PAC system.

Our findings regarding the decrease in performance are in line with the recent work by Fida et al. [21] who analyzed the effect of varying window size from w = 1 s to 3 s and suggests that 1 s to 2 s window size gives a better tradeoff when analyzing static and dynamic activities. On the contrary, more recently Shoaib et al. [22] proposed a system for complex human activity recognition by varying window sizes from 1 s to 30 s and found that increasing window size improves the recognition rate of complex activities. However, our analysis is novel due to the demographics of the studied population. Our work indeed investigates the activities of older adults, whose ADLs may differ from those analyzed by Fida et al. and Shoaib et al. on the younger subjects.

### 3.2. Performance of the PAC Systems in Real-Life Scenarios

#### 3.2.1. In-Lab vs. Out-of-Lab

**Figure 3:**
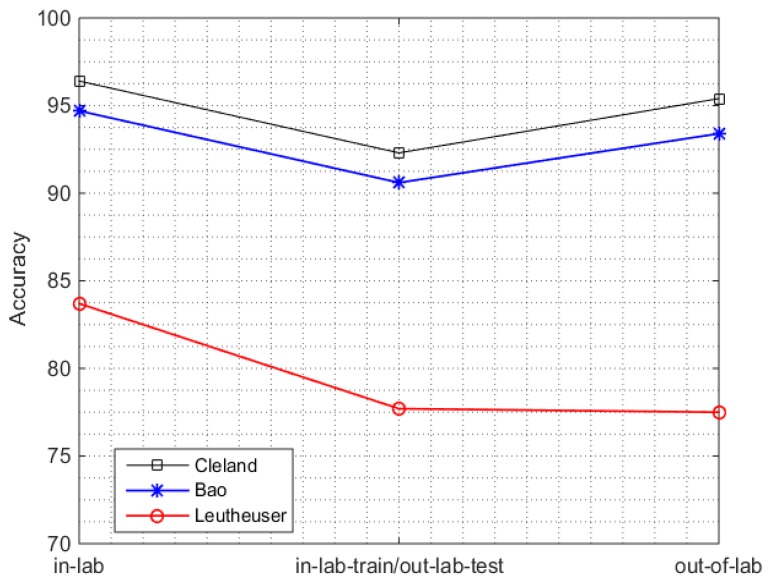
Performance analysis of in-lab, out-of-lab, and in-lab training/out-lab testing scenario for all PAC system using sensor set S_OUT_ (Table 4).

The results of out-of-lab analysis show a decreased accuracy with respect to the in-lab across all systems. Figure 3 (first and last point on time axis), shows the overall accuracy of the three systems in the in-lab and out-of-lab with w = 5 s, chosen as a representative window size. A slight decrease of 1% (96.4%–95.4%) in the work by Cleland et al. and 1.3% (94.7%–93.4%) in the work by Bao et al., is observed. However, such degradation is larger in the work by Leutheuser et al. with a decline of 6.2% (83.7%–77.5%). The best performance of 95.4% is obtained (when trained and tested on the real life data) by the system of Cleland et al. which is quite encouraging, but at the cost of using five sensors and a large features set, which may not be feasible in real-life conditions.

#### 3.2.2. In-Lab Training/Out-Lab Testing

We then evaluated the performance of in-lab trained systems in the real-life setting. In the in-lab training/out-lab testing scenario, the performance of all the SOA systems decreased between 4–6% when compared to the in-lab results (Figure 3). The respective confusion matrix for each SOA system for PAC is shown in Table 6, where sensor set S_OUT_ (Table 4) is used for implementation of all systems. Each sample of the confusion matrix corresponds to a 5s window. Moreover, the accuracies by class and the sensitivities by class for all PAC systems in the in-lab training/out-lab testing scenario are listed in the Table 7. The decreases in accuracy are: from 96.4% to 92.3% (4.1%) in the work by Cleland et al., from 94.7% to 90.6% (4.1%) in the work by Bao et al., and from 83.7% to 77.7% (6.0%) in the work by Leutheuser et al.

The degradation of performance in all the systems in this scenario reflects the lack of field-based validity as highlighted more recently by Lindemann et al. [28]. The reason of this degradation is due the fact that:

(i) Most of the existing PAC systems are developed using a standardized protocol which does not include the ADLs performed under real-life conditions.
(ii) The order and way of performing these activities in a more natural and quite different environment to the one performed in a laboratory environment.

Therefore, these PAC systems are unable to recognize unstructured and unplanned activities in real-life conditions, which emphasizes the urge of developing in-field, validated, PAC systems, as we did when considering the out-of-lab scenario.

Our findings are in-line with the work by Ganea et al. [26], where performance deteriorated when the laboratory-trained system was tested in real life. Our analysis generalizes the fact of performance deterioration over several activities in real life conditions by analyzing sitting, standing, walking, and lying instead of only postural transitions, as analyzed by Ganea et al.

### 3.3. Computational Complexity in the Real-Life Setting

Computational complexity of testing out-of-lab data (when trained on in-lab) is also analyzed by measuring the time required for the feature extraction and for classification (Table 8). The feature computation time is the time required to compute the features of all 16 subjects from out-of-lab data using the sensor set S_OUT_ (Table 4). The testing out-of-lab time, is the total time to test all the out-of-lab data for 16 subjects. Mean and standard deviation of 10 runs (in order to account for computer performance variability) are reported in Table 8. The total window instances obtained (after the feature extraction of the out-of-lab data) for all systems are 36,139 except the system by Leutheuser et al. [10], for which the samples are 35,088 because of the software dependencies. The time consumption analysis of the features computation shows that the time required to compute the features has a direct relationship with the number of sensors. All three systems use multiple sensors and took longer time for feature computation. Moreover, the number of features, and the nature of the features, also plays an important role in computational complexity of the system. For instance, in the work by Leutheuser et al. [10], activity-specific features and hierarchical structure increased the time consumption for the validation. The complexity of the classifier, along with the number of sensors increased the computational time in the systems by Leutheuser et al., and Cleland et al. On the other hand the time taken by Bao et al. is much shorter since it utilizes a simpler classifier approach (decision tree classifier). The computational analysis suggests that in order to make the PAC system operational in real time, optimum number of sensors, proper feature selection to eliminate redundant features, and the choice of simpler and more robust classifier, is very critical. Most of the existing systems do not highlight these factors, especially the selection of features, and of a reduced set of sensors. These factors are crucial for the practical implementation of these systems out of the laboratory.

### 3.4. Performance Comparison of the PAC Systems in the In-Lab Setting Using a Reductionist Approach and Sensitivity Analysis to the Window Size

**Figure 4:**
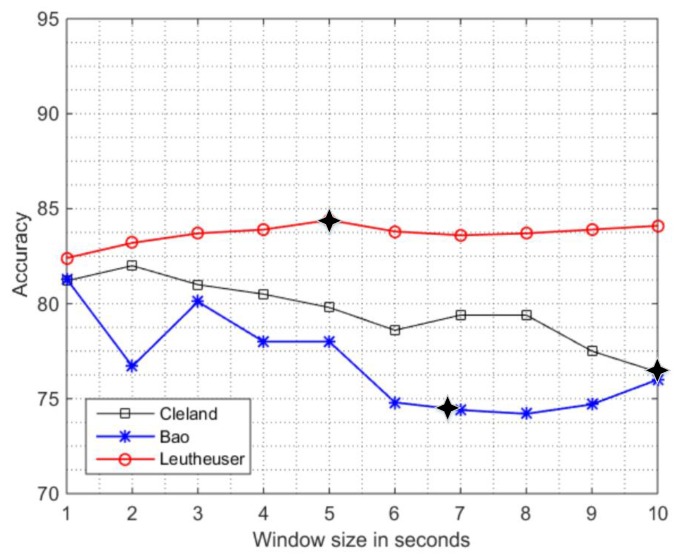
Sensitivity analysis of overall accuracy of in-lab data when window size is increased from w = 1 s to w = 10 s using reductionist approach. The symbol (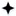) specifies the window size used in the original PAC system by the authors.

The overall performance of the PAC systems using a reductionist approach obtained from the in-lab sensitivity analysis to window size is depicted in Figure 4. In-lab sensitivity analysis using a single sensor at L5 location (Figure 4) follow a decay in performance with the increase in window size (similar to that presented in Section 3.1) for the systems by Bao et al. [2] and Cleland et al. [9]. The deterioration in accuracy from w = 1 s to w = 10 s was 5.3% by Bao et al. and 4.8% by Cleland et al. However, an improvement of 1.7% in accuracy is observed in the work by Leutheuser et al. [10]. In this case, the use of activity specific classification systems instead of using the generalized systems for ADLs seem to be the probable cause.

### 3.5. Performance of the PAC Systems in Real-Life Scenarios Using a Reductionist Approach

#### 3.5.1. In-Lab vs. Out-of-Lab

**Figure 5:**
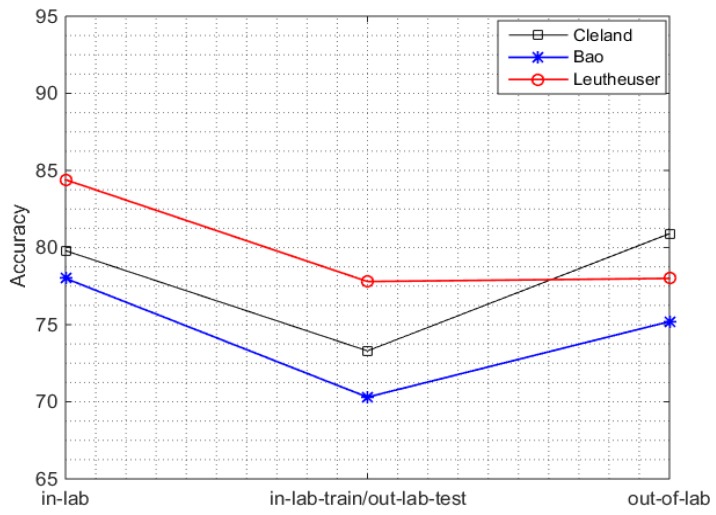
Performance analysis of in-lab, out-of-lab, and in-lab training/out-lab testing scenario for all PAC systems using a reductionist approach.

The analysis using the reductionist approach (Figure 5) shows that accuracy of all systems is decreased except for Cleland et al. in the out-of-lab when compared to in-lab. The decrease is: 2.7% in the work by Bao et al., 6.4% in the work by Leutheuser et al. The slight increase of 1% is observed in the work by Cleland et al. The best performance of 80.9% is achieved by the work of Cleland et al. (similar to Section 3.2.1) when trained and tested on the real-life data which show the potential of using a single sensor in real life conditions. This performance can be enhanced by developing PAC system which incorporates more discriminative features (e.g., biomechanical features) and robust classifier.

#### 3.5.2. In-Lab Training/Out-Lab Testing

The in-lab training/out-lab testing analysis on the single sensing unit also followed the deterioration in overall accuracy and the differences are a bit larger (between 6%–8%) than in the multi-sensor setting (Section 3.2.2) as described by Figure 5. The reduction in the accuracies are: 79.8% to 73.3% (6.5%) by Cleland et al. [9], 84.4% to 77.8% (6.6%) by Leutheuser et al. [10], and 78.0% to 70.3% (7.7%) by Bao et al. [2].

The performance of all systems, both in the original framework and in the reductionist approach degrades for the in-lab testing/out-lab training scenario (when compared to in-lab analysis). Therefore, it is very important to develop a PAC system in the real-life data before releasing it for real life applications, as we did in the out-of-lab analysis. Most of existing system lack this perspective so their performance cannot be generalized for the real life conditions.

## 4. Conclusions

A benchmark study is presented which investigates the performance of various SOA systems for PAC in the in-lab and out-of-lab environment. The sensitivity analysis to window size shows that the increase in window size generally degrades the performance. The in-lab training/out-lab testing analysis concludes that the systems developed in controlled settings are not capable of performing well in real-life conditions where the ADLs are performed in a more natural way. Therefore, the newly-developed systems should be trained and tested on the dataset collected in the real-life conditions. The reductionist approach also obtained similar results for all analyses (in-lab sensitivity analysis to window size, out-of-lab analysis, in-lab training/out-lab testing) but the degradation is much larger than the multi-sensor setup. Furthermore, investigation of the computational complexity is conducted for the feature extraction stage and the classifier testing stage of out-of-lab data. The findings, as we expected, show that the systems with more complex classifier approaches and large numbers of sensors increases the computational complexity of the system.

The number of analyzed subjects (16) is a limitation to overcome in future studies by adding more subjects. However, the analyzed database is one of the largest databases available to date [31], especially considering that the activities were manually annotated with a very high frequency (25 Hz, 25 annotations per second) and this process required significant resources. Another limitation of this study is that it only investigates basic ADLs while real life conditions contain many other activities. 

The reductionist approach we developed which, derived from existing systems, is an important first step to study the effect of reducing the number of sensors in order to find an optimal trade-off between usability and performance (the use of multiple sensors on various body locations can be impractical in real-life).

Our future aim is to develop a physical activity classification system in real life conditions with optimal number of sensors (by exploring various sensor locations), improved feature set (using various feature selection approaches), and robust classification methods to perform comparably to, or better than, existing systems.

## Figures and Tables

**Table 1:** Description of the sensors used for data collection.

Sensor Type	Location	Sampling Frequency	Measured Signals
uSense	Thigh	100 Hz	3D Accelerometer, 3D Gyroscope
uSense	L5	100 Hz	3D Accelerometer, 3D Gyroscope
ActiGraph	Waist	100 Hz	3D Accelerometer
uSense	Chest	100 Hz	3D Accelerometer, 3D Gyroscope
Shimmer	Wrist	200 Hz	3D Accelerometer, 3D Gyroscope
uSense	Feet *	100 Hz	3D Accelerometer, 3D Gyroscope

* Sensor on the feet were not included in out-of-lab data collection.

**Table 2:** In-lab ADLs.

ADL	Total (h)	Occurrences	Mean *	STD *	Min *	Max *	Range *
sitting	1.67	708	8.50	18.90	0.03	267.36	267.33
standing	2.67	1319	7.28	16.40	0.03	296.97	296.94
walking	0.90	613	5.29	2.79	0.96	20.07	19.11
lying	0.28	187	5.47	9.87	0.13	113.23	113.10

* The values are in seconds.

**Table 3:** Out-out-lab ADLs.

ADL	Total (h)	Occurrences	Mean *	STD*	Min *	Max *	Range *
sitting	13.45	497	97.44	200.74	0.04	2075.64	2075.60
standing	6.52	4304	5.45	12.27	0.03	388.52	388.49
walking	4.10	2617	5.64	8.75	0.28	139.56	139.28
lying	0.36	12	106.69	154.02	3.48	583.84	580.36

* The values are in seconds.

**Table 4:** Sensors used from ADAPT dataset to perform the performance on three PAC systems.

Author	S_IN_	S_OUT_
Cleland et al. [9]	Chest, L5, Wrist, Waist, Thigh, Foot	Chest, L5, Wrist, Waist, Thigh
Bao et al. [2]	L5, Wrist, Thigh, Foot	L5, Wrist, Thigh
Leutheuser et al.	Wrist, L5, Chest, Foot	Wrist, L5, Chest

**S_IN_**—Sensors used in our data analysis from In-lab protocol of ADAPT dataset; and **S_OUT_**—Sensors used in our data analysis from out-of- lab protocol of ADAPT dataset.

**Table 5:** Overview of the three SOA systems for PACs implemented in this study for performance analysis.

Author	Fs (W)	S_O_	Experiment Setting (Population)	Features	Activities	Accuracy Reported
Cleland et al. [9]	51.2 (10 s)	Chest, lower back, wrist, hip, thigh, foot	Laboratory setting (8 young adults) (26.25 ± 2.86 years)	Mean, standard deviation, skewness, kurtosis, energy and correlation of axes (separately and average over 3 axes)	Walking, jogging on a treadmill, sitting, lying, standing, walking up stairs, walking down stairs	97.26% SVM
Bao et al. [2]	76.25 (6.7 s)	Hip, wrist, arm, thigh, ankle	Semi-naturalistic conditions (20 subjects) age group not reported	Mean, energy, frequency domain entropy, correlation between the acceleration signals	Walking, sitting, standing, eating or drinking, watching tv, reading, running, bicycling, stretching, strength-training, scrubbing, vacuuming, folding laundry, lying, brushing, climbing stairs, riding elevator, riding escalator	84% using Decision tree
Leutheuser et al. [10]	204.8 (5 s)	Wrist, hip, chest, ankle	Laboratory setting (23 young adults) (27 ± 7 years)	Minimum, maximum, mean and variance, spectral centroid, bandwidth, energy, gravitational component	Sitting, lying, standing, washing dishes, vacuuming, sweeping, walking, running, stairs climbing, bicycling, rope jumping	89.6% hierarchical classifier

Fs—Sampling Frequency in Hz, W = Window Size, **S_O_**—Original set of sensors used by the authors to develop PAC system, Activities—Set of Activities used by authors to develop their PAC system.

**Table 6:** Confusion matrix for the systems; (**a**) Bao et al.; (**b**) Cleland et al.; and (**c**) Leutheuser et al.; in the in-lab training/out-lab testing scenario.

	**(a) Bao et al.**				
	Predicted Class				
**Actual Class**	**stand**	**walk**	**sit**	**lie**	**←classified as**
	9214	571	4	0	stand
	2329	4000	2	9	walk
	24	16	19,260	197	sit
	233	0	2	278	lie
	**(b) Cleland et al.**				
	Predicted Class				
**Actual Class**	**stand**	**walk**	**sit**	**lie**	**←classified as**
	9712	73	4	0	stand
	2474	3857	9	0	walk
	1	1	19,492	3	sit
	0	0	234	279	lie
	**(c) Leutheuser et al.**				
	Predicted Class				
**Actual Class**	**stand**	**walk**	**sit**	**lie**	**←classified as**
	7423	350	1572	16	stand
	395	5397	94	0	walk
	5289	107	13,950	0	sit
	0	0	15	480	lie

**Table 7:** Accuracy and sensitivity by class for all SOA systems for PAC in the in-lab training/out-lab testing scenario.

Authors	Accuracy	Accuracy by Class				Sensitivity by Class			
		Stand	Walk	Sit	Lie	Stand	Walk	Sit	Lie
Bao et al.	90.6	91.3	91.9	99.3	98.8	94.1	63.1	98.8	54.2
Cleland et al.	92.3	92.9	92.9	99.3	99.3	99.2	60.8	100.0	54.4
Leutheuser et al.	77.7	78.3	97.3	79.8	99.9	79.3	91.7	72.1	97.0

**Table 8:** Computational complexity in the in-lab training/out-lab testing scenario.

Author	Feature Computation Mean ± Std (s)	Testing Out-of-Lab Mean ± Std (s)
Bao et al.	337.07 ± 3.10	25.27 ± 0.95
Cleland et al.	458.79 ± 6.57	738.21 ± 1.09
Leutheuser et al.	772.41 ± 11.99	957.83 ± 18.38

## Appendix A. Computation of Accuracy and Sensitivity by Class in the In-Lab Training/Out-Lab Testing Scenario of All SOA for PAC

This section provides the details about the computation of the performance metrics used in this study. The expressions to calculate overall accuracy, accuracy by class, and sensitivity by class are described below:

(A1) $$Accuracy=\frac{TP+TN}{TP+FN+FP+TN}\times 100$$

(A2) $$Ac=\frac{TPc+TNc}{TPc+FNc+FPc+TNc}\times 100$$

(A3) $$Sc=\frac{TPc}{TPc+FNc}\times 100$$

whereas, TP= True Positive, TN = True Negative, FN = False Negative, FP = False Positive.

Ac is the accuracy by class, and Sc is the sensitivity by class. Subscript “c” is used with TP, TN, etc., to represent the metrics by class, for instance, if we are interested in calculating the accuracy and sensitivity of walking activity from the in-lab training/out-lab testing scenario of Bao et al. (Table A1).

**Table A1:** Confusion matrix of the PAC system by Bao et al. in in-lab training/out-lab testing scenario.

Stand	Walk	Sit	Lie	←Classified as
9214	571	4	0	**stand**
2329	4000	2	9	**walk**
24	16	19,260	197	**sit**
233	0	2	278	**lie**

TPc = 4000, FNc = 2340 (2329 + 2 + 9 = 2340), FPc = 587 (571 + 16 + 0 = 587);

TNc = 29,212 (9214 + 4 + 0 + 24 + 19,260 + 197 + 233 + 2 + 278 = 29212)

Therefore:

$$Accuracy=\frac{TP+TN}{TP+FN+FP+TN}=\;\frac{9214+4000+19260+278}{36139\;\left(sum\;of\;all\;instances\right)}\times 100=90.6\%$$

$$Ac=\frac{TPc+TNc}{TPc+FNc+FPc+TNc}=\;\frac{4000+29212}{4000+2340+587+29212}\times 100=91.9\%$$

$$Sc=\frac{TPc}{TPc+FNc}=\;\frac{4000}{4000+2340}\times 100=63.1\%$$

## Appendix B. Detailed Description of the Training and Classification Process Used

This section provides the details about the classifiers used and the training process adapted. The details about the classification procedure and cross-validation procedure are described in Table B1.

The cross-validation process is leave-one-subject-out for the in-lab windowing analysis (trained and tested on in-lab data) and for the out-of-lab analysis (trained and tested on out-of-lab data). The training and testing procedure was different in the in-lab-training/out-lab-testing analysis. In this case, the model was trained using the in-lab data of all subjects, but one, which is being tested on the out-of-lab data.

**Table B1:** Classification procedure used for each PAC system.

Authors	Classifier Used	Cross-Validation Procedure
Cleland et al.	SVM Classifier (with universal Pearson VII function based kernel and complexity value of 100 using WEKA libraries)	Leave-one-subject-out-cross-validation
Bao et al.	Decision Tree Classifier (J48 with default parameters using WEKA libraries)	Leave-one-subject-out-cross-validation
Leutheuseur et al.	Hierarchical Classification (KNN and SVM using WEKA libraries)	Leave-one-subject-out-cross-validation
